# Fibroblast Growth Factor 2 Drives Changes in Gene Expression Following Injury to Murine Cartilage In Vitro and In Vivo

**DOI:** 10.1002/art.38039

**Published:** 2013-08-26

**Authors:** Ka-Wing Chong, Anastasios Chanalaris, Annika Burleigh, Huilin Jin, Fiona E Watt, Jeremy Saklatvala, Tonia L Vincent

**Affiliations:** Kennedy Institute of Rheumatology and University of OxfordLondon, UK

## Abstract

**Objective:**

The articular cartilage is known to be highly mechanosensitive, and a number of mechanosensing mechanisms have been proposed as mediators of the cellular responses to altered mechanical load. These pathways are likely to be important in tissue homeostasis as well as in the pathogenesis of osteoarthritis. One important injury-activated pathway involves the release of pericellular fibroblast growth factor 2 (FGF-2) from the articular cartilage. Using a novel model of murine cartilage injury and surgically destabilized joints in mice, we examined the extent to which FGF-2 contributes to the cellular gene response to injury.

**Methods:**

Femoral epiphyses from 5-week-old wild-type mice were avulsed and cultured in serum-free medium. Explant lysates were Western blotted for phospho-JNK, phospho-p38, and phospho-ERK or were fixed for immunohistochemical analysis of the nuclear translocation of p65 (indicative of NF-κB activation). RNA was extracted from injured explants, rested explants that had been stimulated with recombinant FGF-2 or FGF-18, or whole joints from either wild-type mice or FGF-2^−/−^ mice. Reverse transcription–polymerase chain reaction was performed to examine a number of inflammatory response genes that had previously been identified in a microarray analysis.

**Results:**

Murine cartilage avulsion injury resulted in rapid activation of the 3 MAP kinase pathways as well as NF-κB. Almost all genes identified in murine joints following surgical destabilization were also regulated in cartilage explants upon injury. Many of these genes, including those for activin A (*Inhba*), tumor necrosis factor–stimulated gene 6 (*Tnfaip6*), matrix metalloproteinase 19 (*Mmp19*), tissue inhibitor of metalloproteinases 1 (*Timp1*), and podoplanin (*Pdpn*), were significantly FGF-2 dependent following injury to cartilage in vitro and to joint tissues in vivo.

**Conclusion:**

FGF-2–dependent gene expression occurs in vitro and in vivo in response to cartilage/joint injury in mice.

Articular cartilage is a highly mechanosensitive tissue that responds rapidly to mechanical loading and injury. Over the last decade, we have characterized the early signaling events that occur upon explantation (cutting cartilage from the intact joint surface) and recutting (cutting cartilage that has been equilibrated in vitro in serum-free medium for at least 24 hours prior to injury). Such studies have revealed that explantation leads to activation of the 3 MAP kinases JNK, p38, and ERK (1), as well as activation of NF-κB (1), phosphatidylinositol 3-kinase (PI3K) (Watt F, et al: unpublished observations), and the Src kinases (2). Recutting rested cartilage activates only ERK and PI3K, indicating that the full inflammatory response following cartilage damage can only be realized if the intact joint surface is injured. Other investigators have also studied the molecular response of cartilage to injury and have shown that recutting leads to the cytoplasmic accumulation of β-catenin and the up-regulation of Wnt-dependent genes (3,4).

There are several mechanisms by which injury might drive intracellular signaling in chondrocytes, including through integrins, cell-surface ion channels, purinergic receptors, and the primary cilium (5–10). One mechanism that we have characterized is through the release of fibroblast growth factor 2 (FGF-2), which is stored in the pericellular matrix of cartilage and is liberated upon mechanical stimulation (11–13). FGF-2 release leads to activation of ERK and PI3K upon both explantation and recutting injury. In vivo, FGF-2 is chondroprotective, as mice deficient in FGF-2 develop accelerated spontaneous and surgically induced osteoarthritis (OA) (14). Moreover, injection of FGF-2 into destabilized murine joints delays the development of OA (15). The cellular mechanisms by which FGF-2 is protective in the joint are not fully appreciated, but in vitro and in vivo, FGF-2 is able to suppress the key aggrecan-degrading enzyme ADAMTS-5 (14,16) and induce a number of genes with putative anticatabolic and repair-like activities, including tissue inhibitor of metalloproteinases 1 (TIMP-1) (13) and activin A (17). The activation of inflammatory signaling pathways upon explantation of cartilage is not driven by FGF-2, although our in vitro data suggest that FGF-2 may be able to modulate inflammatory MAP kinase and NF-κB signaling following explantation injury (2).

We recently demonstrated a rapid induction of inflammatory response genes in the joints of mice subjected to acute surgical destabilization (18). The genes for that study were identified from a microarray analysis of RNA extracted from whole joints, and the most highly regulated genes were validated by reverse transcription–polymerase chain reaction (RT-PCR) using custom-designed TaqMan microfluidic cards. They included a number of inflammatory cytokines, such as interleukin-1β (IL-1β) and IL-6, proteases, including ADAMTS-4, ADAMTS-5, and matrix metalloproteinase 3 (MMP-3), chemokines, such as CCL2, CCL5, and CCL7, and a number of putative antiinflammatory/repair genes, such as tumor necrosis factor–stimulated gene 6 (TSG-6) and activin A (18).

Gene induction was extremely rapid following joint destabilization, occurring within 4 hours, and was highly mechanosensitive. Gene responses were largely abrogated if the mouse was paralyzed following surgical joint destabilization. Curiously, if the joint was immobilized by sciatic neurectomy (forcing the knee into rigid extension but allowing weight bearing), only select gene responses were abrogated. Collectively, these data suggested that there are at least 2 different mechanosensing pathways activated in joint tissues following destabilization, each of which drives different sets of genes. We speculated that the pathways that drive mechanosensitive gene regulation following acute joint destabilization in vivo might be the same as those activated by cartilage injury in vitro.

To establish whether the genes induced by joint injury in vivo are the same as those activated in vitro following cartilage injury, we developed a novel cartilage injury model in the mouse, involving avulsion of the immature cartilaginous femoral head. By performing this model using tissue deficient in FGF-2 (hip joint cartilage from FGF-2^−/−^ mice), we determined the relative contribution of this cytokine to the gene expression profile upon cartilage injury in vitro. Finally, we determined the FGF-2 dependence of gene expression in vivo by studying the same gene profiles in the joints of wild-type mice and FGF-2^−/−^ mice following acute joint destabilization.

## MATERIALS AND METHODS

### Animals

Animal experiments were performed after obtaining ethical and statutory approval in accordance with local policy. Mice were kept in an approved animal care facility and were housed 4–6 per cage in standard, individually ventilated cages. C57BL/6J mice ages 4 weeks (for hip avulsion experiments) or 9 weeks (for surgical joint destabilization) were obtained from Charles River. FGF-2^−/−^ mice were originally obtained from The Jackson Laboratory and were backcrossed onto a C57BL/6J background (9 generations).

### Avulsion of hip cartilage

Mice (5–6 weeks old) were culled by cervical dislocation, and the acetabulofemoral joint was exposed by blunt dissection. The hip joint was dislocated by applying pressure from behind the femur, and the femoral cap (containing chondrocytes of the articular surface, secondary ossification center, and growth plate) was avulsed using forceps, as described previously (19). Hip joint cartilage used for RNA extraction were immediately snap frozen (time 0) or were cultured in serum-free medium for up to 4 hours and were then snap frozen. Hip joint cartilage samples used for Western blotting were lysed. Some explants (4–6 per experimental data point) were rested for 48 hours in serum-free medium and then stimulated with recombinant FGF-2 or FGF-18 (PeproTech).

### Surgical joint destabilization

Male mice 10–12 weeks old (3–6 mice per group) were anesthetized by inhalation of isoflurane. All animals received subcutaneous buprenorphine HCl (Vetergesic; Alstoe Animal Heath) postsurgery. Mice were fully mobile within a few minutes following withdrawal of isoflurane. Induction of OA by destabilization of the medial meniscus (DMM) was performed as previously described (14,20). Unoperated (naive) mice were used to normalize the gene expression data.

### Western blotting

Murine cartilage hip explants (3 for MAP kinases and 5 for arginase 1) were lysed in 160 μl of lysis buffer supplemented with a protease cocktail inhibitor (1:100 dilution; Sigma-Aldrich). After 2 hours, the lysates were concentrated using a sodium dodecyl sulfate–polyacrylamide gel electrophoresis (SDS-PAGE) cleanup kit (GE Healthcare/Amersham Biosciences). Samples were resolved on 12% SDS-PAGE gels. Transferred membranes were Western blotted for phospho-JNK (1:1,000 dilution; Cell Signaling Technology catalog no. 9251), phospho-p38 (1:1,000 dilution; Cell Signaling Technology catalog no. 9211), phospho-ERK (1:1,000 dilution; Sigma-Aldrich catalog no. 8159), total ERK (1:1,000 dilution; Santa Cruz Biotechnology catalog no. sc-94), and arginase 1 (1:1,000 dilution; Santa Cruz Biotechnology catalog no. sc-18354).

### Confocal microscopy

Murine hip cartilage explants (n = 3) were snap frozen in liquid nitrogen and embedded in OCT embedding matrix (CellPath). Sections were cut using a cryostat (CM-1900; Leica Microsystems), air dried for 1 hour at room temperature, and then fixed for 20 minutes in 4% (volume/weight) paraformaldehyde in phosphate buffered saline (PBS). Tissue was permeabilized with 0.2% (v/v) Triton X-100, blocked with 10% (v/v) goat serum (DakoCytomation), and incubated overnight at 4°C with a primary antibody against p65 (1:100 dilution). The following day, they were washed and incubated for 2 hours in the dark with Alexa Fluor 488–conjugated fluorescent antibody (Invitrogen) and propidium iodide (1:500 dilution). Fluorescence was visualized using confocal microscopy (Nikon Eclipse TE-2000U) and Volocity Acquisition imaging software (PerkinElmer).

### Quantification of translocation

Three gradient images from each zone (superficial zone [articular cartilage surface], middle zone, and epiphyseal line) were obtained. Translocation was deemed to have occurred if >50% of the nucleus expressed a yellow signal (merging of the red and green fluorescence). The total number of nuclei showing such p65 nuclear translocation was counted for each zone and section and was expressed as a percentage of the total number of nuclei present.

### FGF-2/perlecan staining

Frozen sections of cartilage were air-dried for 1 hour at room temperature and fixed for 10 minutes in 100% methanol. Sections were preincubated for 2 hours at 37°C with chondroitinase ABC (0.1 unit/10 μg), blocked for 1 hour at room temperature with 10% goat serum in PBS, and incubated overnight at 4°C with primary antibodies against FGF-2 (1:1,000 dilution) and the heparan sulfate proteoglycan perlecan (1:1,000 dilution). Sections were washed and then incubated for 1 hour in the dark with secondary antibodies (conjugated with Alexa Fluor 488 for FGF-2 and with Alexa Fluor 647 for perlecan) and propidium iodide (for detection of nuclei). Mounted slides were visualized as above.

### RNA extraction and RT-PCR for whole joints and hip cartilage

Four murine cartilage explants were snap frozen and stored at −80°C. For whole joints, the joint was harvested, and the skin and muscle were removed as previously described (18). The cartilage explants/joints were pulverized using a PowerGen 125 Polytron instrument (Fisher Scientific), and RNA was isolated using RNAqueous lysis buffer (AM1911; Ambion). TRIzol reagent (Helena Biosciences) was used to extract messenger RNA (mRNA), which was then purified using an RNeasy Mini kit (Qiagen). RNA was eluted from the spin columns with 30 μl of nuclease-free water (Promega) and stored at –80°C. RNA quality and concentration were checked by electrophoresis using an Agilent Bioanalyzer with an Agilent RNA 6000 Nano LabChip kit. Only samples with an RNA integrity number ≥6 were used for RT-PCR.

### RT-PCR analysis

RNA was reverse transcribed, and real-time PCR was performed using an ABI Prism 7900HT sequence detection system (SDS 2.2; Applied Biosystems) on 384 custom-made TaqMan microfluidic cards, according to the manufacturer's instructions. A full list of gene accession numbers can be found in the previously published supplementary data (18).

### Determination of arginase 1 activity

Arginase converts arginine to ornithine and urea. The assay measures the production of urea, as previously described (21). Briefly, 0.2 gm of porcine metacarpophalangeal cartilage was lysed in 200 μl of lysis buffer. A total of 160 μl of clarified lysate was mixed with 100 μl of reaction mixture and incubated for 10 minutes at 55°C to activate arginase. The concentration of urea was determined by measuring the absorbance at 540 nm against a standard curve for urea (GE Healthcare/Amersham Biosciences). Samples were read using a Multiskan Biochromatic instrument (Labsystems) and analyzed using Ascent software (Thermo Fisher Scientific).

### Measurement of activin

Secreted activin A protein was quantified using an enzyme-linked immunosorbent assay (ELISA) activin A kit (DuoSet; R&D Systems). Murine cartilage explants were cultured in 60 μl of serum-free Dulbecco's modified Eagle's medium for specified periods of time following injury. A total of 100 μl of standards or samples was assayed in duplicate. Plates were read as above using a spectrophotometric plate reader.

### Statistical analysis

All statistical analyses were performed using GraphPad Prism and Microsoft Excel software. Unpaired *t*-tests were used to compare 2 different groups of samples. One-way analysis of variance (ANOVA) with the Bonferroni post hoc test was used to compare 3 or more groups. Two-way ANOVA with the Bonferroni post hoc test was used to compare 2 variables in 2 or more groups.

## RESULTS

### Activation of MAP kinases and NF-κB upon murine cartilage injury

**Figure 1:**
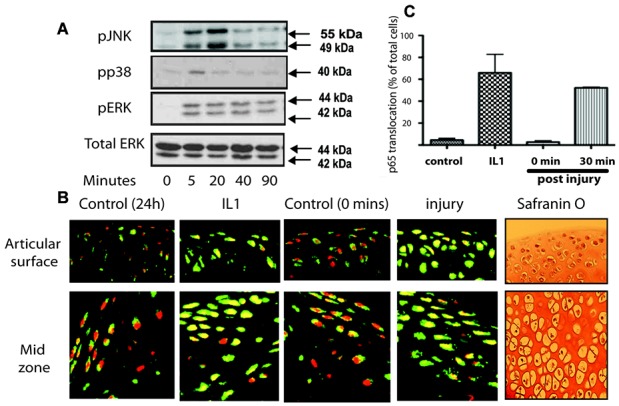
Activation of MAP kinase and NF-κB upon injury of murine cartilage. Three murine hip explants were avulsed onto dry ice (time 0) or into serum-free medium for up to 90 minutes. For some experiments, interleukin-1 (IL-1) was used as a positive control. A, Lysates were prepared and then Western blotted for phosphorylated ERK, p38, and JNK, as well as total ERK (loading control). B, Some explants were snap frozen, embedded in OCT, sectioned, and the sections were stained for visualization of p65 (a component of the NF-κB pathway) (green) and propidium iodide (for nuclei) (red). Signal was detected with fluorescence-tagged secondary antibodies and visualized with confocal microscopy. Also shown are Safranin O–stained sections taken from adjacent regions of the tissue. C, Three separate gradient images were taken from 3 different regions of the explant (superficial zone, middle zone, and epiphyseal line). The percentage of cells demonstrating translocation of p65 (nuclear signal changing from red to yellow) was expressed as a percentage of the total number of nuclei. Values are the mean ± SD. *P* < 0.001 for the comparison of IL-1 versus control as well as for 30 minutes versus 0 minutes.

We previously demonstrated a rapid activation of the 3 MAP kinases and NF-κB upon porcine cartilage explantation (1,13). In the present study, we first determined whether avulsion of the cartilaginous hip joint of 5-week-old mice also produced a similar activation of intracellular signaling pathways. A total of 3 hip cartilage samples per time point were explanted directly onto dry ice (time 0) or were cultured for 5, 20, 40, or 90 minutes in serum-free medium. Lysates were concentrated and immunoblotted for phosphorylated MAP kinases. Figure 1A shows the activation of the 3 MAP kinases (phospho-JNK, phospho-p38, and phospho-ERK) upon hip explant avulsion injury. A transient activation of p38 and JNK occurred with sustained activation of the ERK pathway.

To determine whether there was activation of the NF-κB pathway, we measured the translocation of the p65 NF-κB subunit from the cytoplasm to the nucleus by confocal microscopy upon IL-1 stimulation (positive control) or upon injury. Nuclear translocation of p65 (nuclear staining changing from red to yellow) was apparent through the depths of the tissue at 30 minutes following IL-1 stimulation of rested (24 hours postavulsion) hip cartilage. A similar p65 translocation was also apparent 30 minutes after avulsion injury alone (Figure 1B). Results were quantified and expressed as the percentage of cells in which nuclear translocation had occurred (Figure 1C).

### Gene expression profiles following murine cartilage injury

We have recently shown that there is rapid (4 hours) induction of inflammatory response genes upon surgical joint destabilization in 10-week-old mice (18). The genes were determined to be highly mechanosensitive, as joint immobilization (by anesthetic or by sciatic neurectomy) suppressed many of the responses. Although the initial analysis was performed on RNA extracted from whole joints, we also observed that the same genes (with just a couple of exceptions) were up-regulated within the articular cartilage (18). We sought to determine whether cartilage explantation (avulsion) injury in vitro would induce a similar profile of genes as that seen in the articular cartilage in response to joint destabilization in vivo.

Four hip cartilage explants were avulsed onto dry ice (time 0) or were cultured for 4 hours in serum-free medium. RNA was extracted according to a newly established protocol (see Materials and Methods), and RT-PCR was performed using the custom-designed TaqMan microfluidic cards that were used in our previous in vivo study. Table1 shows an abridged list containing 18 genes, 17 of which were regulated in the joint following surgical destabilization (18).

**Table 1:** Activation of inflammatory response genes upon avulsion injury in murine cartilage explants*

Gene	Gene induction in hip joint cartilage 4 hours after injury	*P*
*Arg1*	112.81 ± 93.58	≤0.05
*Ccl2*	12.39 ± 7.86	≤0.01
*Il6*	48.01 ± 30.49	≤0.01
*Mmp3*	3.28 ± 0.95	≤0.001
*Il1b*	3.67 ± 1.34	≤0.05
*Adamts1*	2.45 ± 1.0	≤0.01
*Adamts4*	1.25 ± 0.53	NS
*Adamts5*	2.01 ± 0.42	≤0.001
*Tnfaip6* (*Tsg6*)	39.86 ± 22.10	≤0.01
*Wisp2*	0.52 ± 0.23 (↓)	≤0.001
*Tnfrsf12a* (*Tweak*)	11.68 ± 6.72	≤0.01
*Ptgs2* (*Cox2*)	15.68 ± 8.47	≤0.001
*Pdpn*	5.89 ± 1.34	≤0.001
*Timp1*	4.61 ± 1.04	≤0.001
*Cd14*	16.38 ± 6.69	≤0.001
*Mmp19*	3.43 ± 1.76	≤0.01
*Inhba*	98.74 ± 56.61	≤0.001
*Cd68*	0.18 ± 0.10 (↓)	≤0.001

* Hip cartilage explants from 5-week-old mice were avulsed and cultured for 4 hours in serum-free medium. RNA was extracted (4 cartilage samples pooled for each experimental data point), and reverse transcription–polymerase chain reaction was performed for a number of genes previously identified from a microarray study. Gene expression was normalized to the 18S housekeeping gene and expressed relative to the levels at time 0. Only 2 genes, *Wisp2* and *Cd68*, were found to be down-regulated. Values are the mean ± SD fold change from time 0 (n = 6 experimental data points per group). *P* values were determined by unpaired *t*-test. NS = not significant.

The complete list of 45 genes examined is shown in Supplementary Table1 (available on the *Arthritis & Rheumatism* web site at http://onlinelibrary.wiley.com/doi/10.1002/art.38039/abstract). Of the 43 genes examined following joint destabilization, 35 of them were regulated in the whole joint following surgery (significance denoted by *P* value in parentheses). Of these, 25 (71%) were also significantly regulated in hip cartilage upon injury in vitro. Some genes were strongly down-regulated by in vitro cartilage injury (MMP-8, ADAMTS-15, estrogen receptor 1, androgen receptor, CCR2, and CD68). This most likely reflected a loss of cells expressing these molecules from the explants during culture (most likely, immune cells of the vascular growth plate). These genes were therefore not included in further analyses.

**Figure 2:**
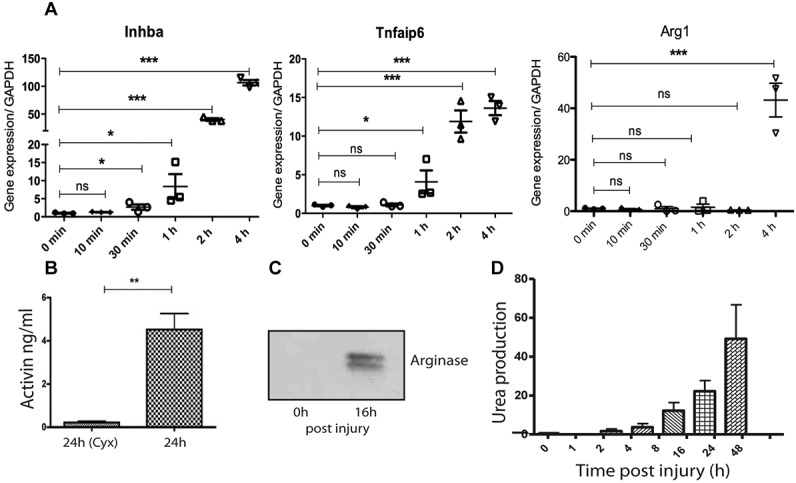
Rapid induction of inflammatory response genes following injury of murine cartilage. A, Hip cartilage explants were snap frozen or were cultured for various times (up to 48 hours) postavulsion in serum-free medium. RNA was extracted, and reverse transcription–polymerase chain reaction analysis of inhibin A, tumor necrosis factor–stimulated gene 6, and arginase 1 was performed. Results are expressed relative to GAPDH. B, For protein verification, conditioned medium from injured explants was analyzed for activin A levels by enzyme-linked immunosorbent assay. Explants cultured in cycloheximide (Cyx) for the same period (24 hours) were used as a negative control. C, Lysates from immediately lysed cartilage (0 hours) or from cultured explants (16 hours) were Western blotted for arginase 1. D, Arginase 1 activity was assayed by measuring the production of urea from L-arginine in the presence of cartilage explant lysates taken at different times following injury. Values in A, B, and D are the mean ± SD; each symbol in A represents a single experimental data point. ∗ = *P* < 0.05; ∗∗ = *P* < 0.01; ∗∗∗ = *P* < 0.001. NS = not significant.

Figure 2 shows examples of 3 genes, inhibin βA (the subunit of activin A), TSG-6 (*Tnfaip6*), and arginase 1, and their induction over the first 4 hours following cartilage injury. Of note, some genes, such as inhibin βA, were rapidly induced postinjury, (first appearing at 30 minutes), while others, such as arginase 1, were regulated only at 4 hours postinjury (Figure 2A). We confirmed protein expression for 2 of the genes: inhibin βA and arginase 1. Inhibin βA homodimerizes to form transforming growth factor β (TGFβ) family member activin A. We have previously shown that this molecule is released by OA tissue (22), and its secretion following cartilage injury has been shown to be dependent upon FGF-2 release (17). We explanted hips into serum-free medium or into medium containing cycloheximide to inhibit new protein synthesis (negative control). Medium was collected for 24 hours following injury, and secreted activin A was measured by ELISA. Activin A was strongly induced upon injury and was dependent upon new protein synthesis (Figure 2B). Arginase 1 was also detected at the protein level by Western blotting of chondrocyte lysates (Figure 2C), and its activity was assessed using an assay that measures the production of urea, a product of the activity of arginase on its substrate L-arginine. Arginase activity increased significantly from 4 hours to 48 hours postinjury (Figure 2D).

### FGF-2 dependence of gene induction upon cartilage injury in vitro and in vivo

The advantage of performing cartilage injury studies in murine tissue is that such studies can be performed in genetically modified tissues to determine the relative contribution of a given molecule to the injury response. One molecule of interest is FGF-2, as we have shown that it is released from cartilage upon injury and is able to change chondrocyte gene expression in vitro (13). We first checked whether the genes that were regulated by injury were also induced by exogenous recombinant FGF-2. Murine hip explants were avulsed and then rested in serum-free medium for 48 hours, a time that was chosen because it was the point at which the induction of most genes had returned to basal levels following avulsion injury. However, at 48 hours, 3 genes were still elevated above the levels at time 0 (immediately following avulsion). These were arginase 1, CCL2, and podoplanin, which were up-regulated 267-fold, 8.1-fold, and 5.2-fold, respectively, compared to time 0 (data not tabulated).

**Figure 3:**
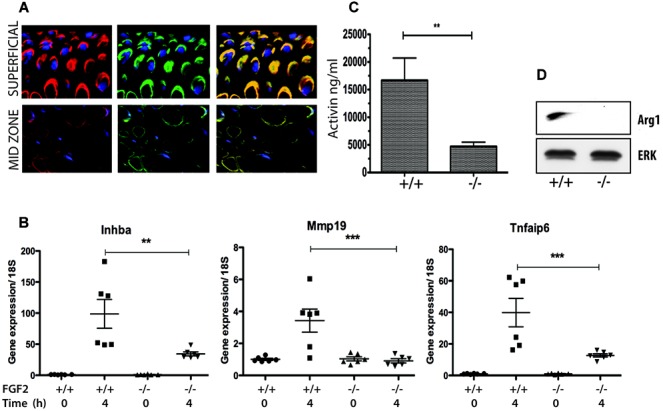
Induction of fibroblast growth factor 2 (FGF-2)–dependent genes following injury of murine cartilage. A, Confocal microscopy of hip cartilage explants from wild-type mice was performed to determine the pericellular location of perlecan (red) and FGF-2 (green). Note the colocalization of perlecan and FGF-2 through the entire tissue depth. B, For gene expression analyses, RNA was extracted at 0 hours or 4 hours postinjury from 4 pooled hip cartilage explants obtained from wild-type mice and from FGF-2^−/−^ mice (n = 6 mice per group). Values were normalized to 18S and expressed relative to time 0. C and D, FGF-2–dependent protein measurements of activin (C) and arginase 1 (D) were determined by enzyme-linked immunosorbent assay and Western blotting, respectively. Activin was measured in medium collected over 24 hours from single-injured explants of hip joint cartilage from wild-type mice (+/+) and FGF-2^−/−^ mice (n = 3 per group). Arginase 1 and total ERK (loading control) were measured by Western blotting of lysates from injured explants (24 hours) (n = 3 pooled explants). Values in B and C are the mean ± SD. ∗∗ = *P* < 0.01; ∗∗∗ = *P* < 0.005.

Following FGF-2 stimulation of rested explants, 7 of 16 genes were found to be significantly elevated (Table2). These included inhibin βA, TIMP-1, and prostaglandin-endoperoxide synthase 2 (PTGS-2), which we previously observed to be FGF-2 dependent. To confirm the FGF-2 dependence of gene regulation upon explantation injury, we next explanted cartilage from either wild-type (C57BL/6J) mice or FGF-2^−/−^ mice into serum-free medium for 4 hours. Because hip explants contain a mixture of chondrocytes (those from the articular surface as well as those destined to become the secondary ossification center), we first determined the presence of pericellular FGF-2 within the hip explant. Figure 3A shows images from confocal microscopy of hip explants stained for perlecan (to delineate the pericellular matrix) and FGF-2. The results indicated that in hip joints from wild-type mice, an FGF-2–rich pericellular matrix was apparent throughout all the cells of the femoral head.

**Table 2:** FGF-2 dependence of inflammatory response genes in vitro

Gene	Gene expression following FGF-2 stimulation of rested cartilage*		Gene expression in FGF-2^−/−^ mouse cartilage 4 hours postavulsion†	Gene expression ratio in FGF-2^−/−^ mice versus wild-type mice‡	
	Wild-type mice	*P*		Ratio	*P*
*Arg1*	2.0 ± 1.5	NS	22.77 ± 17.98	0.2	≤0.01
*Ccl2*	5.9 ± 2.1	NS	33.46 ± 42.14	2.7	NS
*Il6*	7.8 ± 4.8	NS	232.57 ± 109.22	4.8	NS
*Mmp3*	7.2 ± 5.8	NS	2.58 ± 1.46	0.8	NS
*Il1b*	8.1 ± 6.7	NS	21.19 ± 24.95	5.7	NS
*Adamts1*	2.7 ± 2.3	NS	3.00 ± 0.95	1.2	NS
*Adamts4*	2.1 ± 0.9	NS	1.36 ± 0.43	1.1	NS
*Adamts5*	1.8 ± 1.7	NS	1.75 ± 0.44	0.9	NS
*Tnfaip6* (*Tsg6*)	20 ± 8.6	≤0.01	12.72 ± 2.49	0.3	≤0.001
*Tnfrsf12a* (*Tweak*)	15 ± 5.7	≤0.05	7.61 ± 3.59	0.7	≤0.05
*Ptgs2* (*Cox2*)	15 ± 5.5	≤0.01	16.19 ± 5.65	1.0	NS
*Pdpn*	5.0 ± 3.1	≤0.05	3.04 ± 0.95	0.5	≤0.001
*Timp1*	9.2 ± 2.8	≤0.001	3.07 ± 0.49	0.67	≤0.01
*Cd14*	18 ± 8.4	≤0.05	6.76 ± 1.56	0.4	≤0.001
*Mmp19*	1.2 ± 0.7	NS	0.91 ± 0.31	0.3	≤0.001
*Inhba*	31 ± 17	≤0.05	34.43 ± 7.70	0.4	≤0.01

* Hip explants from 5-week-old wild-type mice were avulsed, rested for 48 hours, and then stimulated for 4 hours with recombinant fibroblast growth factor 2 (FGF-2; 20 ng/ml). RNA was extracted (4 cartilage samples pooled for each experimental data point), and reverse transcription–polymerase chain reaction was performed for the same genes as in Table1. Gene expression was normalized to the 18S housekeeping gene and expressed relative to the levels in unstimulated cartilage explants. Values are the mean ± SD of 3 experimental data points per group. NS = not significant (i.e., not significantly FGF-2 dependent following stimulation with exogenous growth factor or endogenous FGF-2).

† Gene expression levels are relative to those at time 0. Values are the mean ± SD of 6 experimental data points per group.

‡ Gene expression levels are from Table1. Lower ratios indicate stronger FGF-2 dependence.

Table2 shows the genes whose expression was significantly blunted in the FGF-2^−/−^ mouse cartilage (a significant decrease in the ratio of gene expression in FGF-2^−/−^ mice versus wild-type mice). These results were largely consistent with those of the FGF-2 stimulation experiment, in that most genes that were regulated by FGF-2 stimulation in vitro were suppressed in cartilage from FGF-2^−/−^ mice upon explantation injury. The exceptions to this included arginase 1 and MMP-19, which were strongly FGF-2 dependent upon explantation but were not regulated by recombinant FGF-2. Conversely, PTGS-2 was strongly regulated by recombinant FGF-2, but gene regulation upon explantation was not altered by the absence of endogenous FGF-2. Some inflammatory genes appeared to be FGF-2 independent, including the ADAMTS genes, IL-1β, and IL-6. Overall, ∼50% of genes were significantly FGF-2 dependent. Three of these, inhibin βA, MMP-19, and TSG-6 (*Tnfaip6*), are shown at the mRNA level in Figure 3B. FGF-2 dependence at the protein level was demonstrated for inhibin βA (activin) and arginase 1 (Figures 3C and D).

As these injury genes were regulated in response to acute joint destabilization, we next determined whether expression of the same genes in vivo was also dependent upon FGF-2. To test this, the joints of wild-type mice and FGF-2^−/−^ mice were surgically destabilized, and whole-joint mRNA was extracted 6 hours later. Table3 shows the genes that were FGF-2 dependent following acute joint destabilization. With the exception of MMP-19, all genes that were found to be FGF-2 dependent following cartilage injury in vitro were also FGF-2 dependent following injury in vivo. In addition, 3 other inflammatory response genes were FGF-2 dependent in vivo: CCL2, IL-6, and MMP-3.

**Table 3:** FGF-2 dependence of inflammatory response genes in vivo following surgical joint destabilization

Gene	Gene regulation 6 hours following DMM*		Gene expression ratio in FGF-2−/− mice versus wild-type mice†		Gene regulation post-DMM suppressed by sciatic neurectomy‡
	Wild-type mice	FGF-2^−/−^ mice	Ratio	*P*	
*Arg1*	98.71 ± 24.0	5.82 ± 4.98	0.05	≤0.005	Yes
*Ccl2*	153 ± 42.30	12.03 ± 3.0	0.08	≤0.05	Yes
*Il6*	21.02 ± 0.42	2.12 ± 0.311	0.10	≤0.001	Yes
*Mmp3*	5.99 ± 0.29	2.89 ± 0.71	0.48	≤0.005	Yes
*Il1b*	5.56 ± 0.41	5.46 ± 1.01	0.98	NS	Yes
*Adamts1*	6.71 ± 4.9	2.43 ± 0.19	0.36	NS	Yes
*Adamts4*	4.48 ± 0.51	4.94 ± 0.46	1.10	NS	Yes
*Adamts5*	1.82 ± 0.22	2.03 ± 0.54	1.11	NS	Yes
*Tnfaip6* (*Tsg6*)	33.83 ± 1.6	2.19 ± 0.47	0.06	≤0.001	No
*Tnfrsf12a* (*Tweak*)	5.76 ± 0.71	1.08 ± 0.13	0.19	≤0.001	No
*Ptgs2* (*Cox2*)	15.60 ± 1.22	19.64 ± 2.32	1.26	NS	No
*Pdpn*	6.95 ± 0.15	3.15 ± 0.96	0.45	≤0.005	No
*Timp1*	5.06 ± 0.18	2.54 ± 0.59	0.50	≤0.005	No
*Cd14*	6.78 ± 0.37	3.59 ± 1.37	0.53	≤0.05	No
*Mmp19*	3.1 ± 0.10	2.68 ± 0.48	0.87	NS	No
*Inhba*	2.55 ± 0.39	0.54 ± 0.11	0.21	≤0.005	No

* RNA was extracted from whole joints obtained from naive (unoperated) mice or from wild-type mice and FGF-2^−/−^ mice following surgical destabilization of the medial meniscus (DMM). Results were normalized to the 18S housekeeping gene and expressed relative to the values in naive mouse joints. Values are the mean ± SD of 6 mice per group.

† Lower ratios indicate stronger fibroblast growth factor 2 (FGF-2) dependence. NS = not significant.

‡ The findings concerning the ability of neurectomy to suppress gene expression in vivo following DMM are from ref.^19^.

## DISCUSSION

We describe herein a novel cartilage injury assay in the mouse that recapitulates injury responses in porcine articular cartilage with regard to activation of intracellular signaling pathways and induction of inflammatory genes. We used this model to investigate a broad range of injury response genes identified originally from a microarray study of acutely destabilized joints (18). Some of these genes, such as activin A (*Inhba*) and TIMP-1, are known to be induced in cartilage following injury, and our previous studies have indicated the FGF-2 dependence of their induction (13,17).

We observed that genes that were induced in the joint following surgical destabilization were also activated upon crude explantation injury of cartilage in vitro. This may not be altogether surprising, since almost all genes that were regulated in whole joints were also regulated specifically in the articular cartilage in vivo (18). Taken together, these findings suggest that there is, in essence, a cartilage injury response that occurs when the joint is destabilized. Similar gene changes also occur within other tissues of the joint, implying that connective tissue injury produces a generic molecular response (18).

One mechanism by which chondrocytes sense tissue injury is through the release of FGF-2 from the pericellular matrix, where it is sequestered on the heparan sulfate chains of the proteoglycan perlecan (11,13). When exogenous FGF-2 was used to stimulate chondrocytes, several of the genes we studied appeared to be FGF-2 dependent. Likewise, when the in vitro injury response was studied using hip explants from FGF-2^−/−^ mice, FGF-2–dependent genes were suppressed, confirming that the release of FGF-2 upon injury significantly contributes to the acute injury response. The most notable exception to this was the strong FGF-2 dependence of arginase 1 expression following explant injury.

Arginase 1 expression was unusual because its regulation following explantation was slow to start (4 hours rather than 30 minutes) and was maximal at ∼24 hours. At 48 hours postexplantation (the time at which the rested explants were stimulated with recombinant FGF-2 in vitro), arginase 1 mRNA was still elevated 267-fold above basal levels. This may explain why it was difficult to measure a discernible regulation of arginase 1 following simple FGF-2 stimulation in vitro. The other possibility is that FGF-2 is able to synergize with other inflammatory signaling pathways to induce inflammatory response genes. This would be consistent with our data showing that explantation of porcine cartilage into the selective FGF receptor 1 (FGFR-1) inhibitor SB402451 (also known as PD173074) suppresses injury-induced NF-κB activity and regulation of inflammatory response genes (2).

A number of genes, including CCL2, IL-6, and MMP-3, appeared to show different FGF-2–dependent responses in vivo and in vitro. These were not significantly induced by FGF-2 in vitro but were FGF-2 dependent following in vivo injury (down-regulated in FGF-2^−/−^ mice). This is most likely explained by the fact that in vivo, other tissues contribute to the gene response, such as the synovium and migrating immune cells. It is possible that the FGF-2 dependence of these genes is more apparent in these noncartilaginous tissues. Some genes, such as IL-1α, IL-1 receptor type I, and CD68, were superinduced in FGF-2^−/−^ mouse joints following DMM (see Supplementary Table1, available on the *Arthritis & Rheumatism* web site at http://onlinelibrary.wiley.com/doi/10.1002/art.38039/abstract). This would be consistent with our unpublished work showing that the level of synovitis is higher in FGF-2^−/−^ mice following DMM. Changes in gene expression in vivo may therefore also relate to the number and type of inflammatory cells that are present in the joint.

FGF-2^−/−^ mice develop accelerated disease following joint destabilization (14), so it follows that the FGF-2–dependent gene responses measured in vitro and in vivo are likely to be neutral for the joint or chondroprotective. Candidate chondroprotective genes include TSG-6 (*Tnfaip6*), a hyaluronan-binding molecule that is antiinflammatory in vivo, possibly by blocking neutrophil extravasation into the inflamed joint (23–25). Activin A (*Inhba*) is a TGFβ family member that has anticatabolic effects in vitro (17), plays antiinflammatory roles (26), and may stimulate tissue repair (27). TIMP-1 is a broad-spectrum metalloproteinase inhibitor, so it potentially has anticatabolic actions in the joint (28).

One striking result observed when these data are considered alongside our previously published data (18) is that the FGF-2–dependent genes largely appear to be the ones that were suppressed in vivo when the joint was completely immobilized (i.e., mechanosensitive) but not when the joint was immobilized by sciatic neurectomy. Neurectomized mice were unable to flex the knee joint because of paralysis of the hamstring muscles but were able to bear weight through the joint. The mice walked with a straight leg, with compensatory flexion at the hip. Taken together, these results suggest that compressive force (weight bearing through a fully extended leg) is sufficient to activate FGF-2–dependent genes. As neurectomized mice were protected from developing OA, this further confirms the neutral or protective role of FGF-2–dependent genes in vivo. In contrast, dynamic flexion of the knee joint induced both FGF-2–dependent and FGF-2–independent genes, suggesting that dynamic flexion of the knee joint activates another mechanosensing pathway that can be modified by FGF-2, which indicates that the type of mechanical joint loading could determine the expression of catabolic and joint-protective genes. Understanding this molecular response to joint destabilization not only identifies possible gene targets for disease, but also potentially helps us to design physical rehabilitation protocols for patients who have experienced an acute joint injury.

One further interesting possibility deserves consideration. Recently published studies have begun to unravel the complex and seemingly contradictory role of FGF-2 in chondrocyte gene expression in vitro by suggesting that responses to FGF-2 may differ according to whether the signal is delivered through FGFR-1 or FGFR-3 (the two most abundant FGF receptors in human articular chondrocytes) (29,30). In vivo, this is supported by the observation that FGFR-3^−/−^ mice develop accelerated OA with age (31) (like the FGF-2^−/−^ mice), but a recently described FGFR-1 conditional-null mouse, where FGFR-1 in the cartilage is deleted postnatally, was strongly protected from OA following joint destabilization (32). We looked to see whether the pattern of gene expression in cartilage was different following stimulation with an FGFR-3–selective ligand (FGF-18) compared to a pan–FGFR ligand (FGF-2). Supplementary Table2 (available on the *Arthritis & Rheumatism* web site at http://onlinelibrary.wiley.com/doi/10.1002/art.38039/abstract) shows that FGF-18 regulated the same genes as FGF-2 in vitro, indicating that FGFR-3 is able to mediate these changes in gene expression. In the absence of an FGFR-1 selective agonist or specific FGFR-knockout mice, it is not possible to establish the relative importance of FGFR-1 with regard to these changes. Interestingly, both FGF-2 and FGF-18 down-regulated FGFR-3 mRNA and up-regulated FGF-18 mRNA.

By combining in vitro studies with tissue samples from genetically modified mice, this study has allowed the molecular contribution of a known injury mediator, FGF-2, to be examined following cartilage damage. Since the same genes are regulated in an FGF-2–dependent manner in vivo following joint destabilization, the results of this study tell us that the pathways induced by crude injury in vitro are relevant to the pathways consequent upon joint destabilization in vivo. More importantly, such in vitro cartilage injury assays are accessible and economical with regard to time and numbers of animals needed and, thus, provide a useful screening tool for the identification and characterization of mechanosensitive pathways.

## References

[1] Gruber J, Vincent TL, Hermansson M, Bolton M, Wait R, Saklatvala J (2004). Induction of interleukin-1 in articular cartilage by explantation and cutting. Arthritis Rheum.

[2] Watt FE, Ismail HM, Didangelos A, Peirce M, Vincent TL, Wait R (2013). Src and fibroblast growth factor 2 independently regulate signaling and gene expression induced by experimental injury to intact articular cartilage. Arthritis Rheum.

[3] Dell'Accio F, De Bari C, Eltawil NM, Vanhummelen P, Pitzalis C (2008). Identification of the molecular response of articular cartilage to injury, by microarray screening: Wnt-16 expression and signaling after injury and in osteoarthritis. Arthritis Rheum.

[4] Dell'Accio F, De Bari C, El Tawil NM, Barone F, Mitsiadis TA, O'Dowd J (2006). Activation of WNT and BMP signaling in adult human articular cartilage following mechanical injury. Arthritis Res Ther.

[5] Wann AK, Zuo N, Haycraft CJ, Jensen CG, Poole CA, McGlashan SR (2012). Primary cilia mediate mechanotransduction through control of ATP-induced Ca^2+^ signaling in compressed chondrocytes. FASEB J.

[6] Leong DJ, Hardin JA, Cobelli NJ, Sun HB (2011). Mechanotransduction and cartilage integrity. Ann N Y Acad Sci.

[7] Spiteri C, Raizman I, Pilliar RM, Kandel RA (2010). Matrix accumulation by articular chondrocytes during mechanical stimulation is influenced by integrin-mediated cell spreading. J Biomed Mater Res A.

[8] Mobasheri A, Lewis R, Maxwell JE, Hill C, Womack M, Barrett-Jolley R (2010). Characterization of a stretch-activated potassium channel in chondrocytes. J Cell Physiol.

[9] Garcia M, Knight MM (2010). Cyclic loading opens hemichannels to release ATP as part of a chondrocyte mechanotransduction pathway. J Orthop Res.

[10] Ramage L, Nuki G, Salter DM (2009). Signalling cascades in mechanotransduction: cell-matrix interactions and mechanical loading. Scand J Med Sci Sports.

[11] Vincent TL, McLean CJ, Full LE, Peston D, Saklatvala J (2007). FGF-2 is bound to perlecan in the pericellular matrix of articular cartilage, where it acts as a chondrocyte mechanotransducer. Osteoarthritis Cartilage.

[12] Vincent T, Saklatvala J (2006). Basic fibroblast growth factor: an extracellular mechanotransducer in articular cartilage?. Biochem Soc Trans.

[13] Vincent T, Hermansson M, Bolton M, Wait R, Saklatvala J (2002). Basic FGF mediates an immediate response of articular cartilage to mechanical injury. Proc Natl Acad Sci U S A.

[14] Chia SL, Sawaji Y, Burleigh A, McLean C, Inglis J, Saklatvala J (2009). Fibroblast growth factor 2 is an intrinsic chondroprotective agent that suppresses ADAMTS-5 and delays cartilage degradation in murine osteoarthritis. Arthritis Rheum.

[15] Li X, Ellman MB, Kroin JS, Chen D, Yan D, Mikecz K (2012). Species-specific biological effects of FGF-2 in articular cartilage: implication for distinct roles within the FGF receptor family. J Cell Biochem.

[16] Sawaji Y, Hynes J, Vincent T, Saklatvala J (2008). Fibroblast growth factor 2 inhibits induction of aggrecanase activity in human articular cartilage. Arthritis Rheum.

[17] Alexander S, Watt F, Sawaji Y, Hermansson M, Saklatvala J (2007). Activin A is an anticatabolic autocrine cytokine in articular cartilage whose production is controlled by fibroblast growth factor 2 and NF-κB. Arthritis Rheum.

[18] Burleigh A, Chanalaris A, Gardiner MD, Driscoll C, Boruc O, Saklatvala J (2012). Joint immobilization prevents murine osteoarthritis and reveals the highly mechanosensitive nature of protease expression in vivo. Arthritis Rheum.

[19] Stanton H, Golub SB, Rogerson FM, Last K, Little CB, Fosang AJ (2011). Investigating ADAMTS-mediated aggrecanolysis in mouse cartilage. Nat Protoc.

[20] Glasson SS, Chambers MG, van den Berg WB, Little CB (2010). The OARSI histopathology initiative—recommendations for histological assessments of osteoarthritis in the mouse. Osteoarthritis Cartilage.

[21] Imagama T, Ogino K, Takemoto K, Kato Y, Kataoka H, Suzuki H (2012). Regulation of nitric oxide generation by up-regulated arginase I in rat spinal cord injury. J Clin Biochem Nutr.

[22] Hermansson M, Sawaji Y, Bolton M, Alexander S, Wallace A, Begum S (2004). Proteomic analysis of articular cartilage shows increased type II collagen synthesis in osteoarthritis and expression of inhibin βA (activin A), a regulatory molecule for chondrocytes. J Biol Chem.

[23] Bardos T, Kamath RV, Mikecz K, Glant TT (2001). Antiinflammatory and chondroprotective effect of TSG-6 (tumor necrosis factor-α-stimulated gene-6) in murine models of experimental arthritis. Am J Pathol.

[24] Szanto S, Bardos T, Gal I, Glant TT, Mikecz K (2004). Enhanced neutrophil extravasation and rapid progression of proteoglycan-induced arthritis in TSG-6–knockout mice. Arthritis Rheum.

[25] Wisniewski HG, Hua JC, Poppers DM, Naime D, Vilcek J, Cronstein BN (1996). TNF/IL-1-inducible protein TSG-6 potentiates plasmin inhibition by inter-α-inhibitor and exerts a strong antiinflammatory effect in vivo. J Immunol.

[26] De Kretser DM, O'Hehir RE, Hardy CL, Hedger MP (2012). The roles of activin A and its binding protein, follistatin, in inflammation and tissue repair. Mol Cell Endocrinol.

[27] Jazwinska A, Badakov R, Keating MT (2007). Activin-βA signaling is required for zebrafish fin regeneration. Curr Biol.

[28] Troeberg L, Nagase H (2012). Proteases involved in cartilage matrix degradation in osteoarthritis. Biochim Biophys Acta.

[29] Vincent TL (2011). Fibroblast growth factor 2: good or bad guy in the joint?. Arthritis Res Ther.

[30] Yan D, Chen D, Cool SM, van Wijnen AJ, Mikecz K, Murphy G (2011). Fibroblast growth factor receptor 1 is principally responsible for fibroblast growth factor 2-induced catabolic activities in human articular chondrocytes. Arthritis Res Ther.

[31] Valverde-Franco G, Binette JS, Li W, Wang H, Chai S, Laflamme F (2006). Defects in articular cartilage metabolism and early arthritis in fibroblast growth factor receptor 3 deficient mice. Hum Mol Genet.

[32] Weng T, Yi L, Huang J, Luo F, Wen X, Du X (2012). Genetic inhibition of fibroblast growth factor receptor 1 in knee cartilage attenuates the degeneration of articular cartilage in adult mice. Arthritis Rheum.

