# Supplementary material for: Fibroblast Growth Factor 2 Drives Changes in Gene Expression Following Injury to Murine Cartilage In Vitro and In Vivo
Source: Arthritis Rheum. 2013 Aug 26;65(9):2346–55. doi: 10.1002/art.38039 (PMC3992838; doi:10.1002/art.38039)
Supplement: Supplementary file 1 [file art0065-2346-sd1.docx]

| **Supplementary table 1.** FGF2 dependent genes on cartilage injury in vitro and in vivo | | | | | | | | |
| --- | --- | --- | --- | --- | --- | --- | --- | --- |
| ***GENE*** | **IN VITRO GENE RESPONSES (cartilage injury)** | | | | **IN VIVO GENE RESPONSES (whole joint)** | | | |
|  | ***4h versus 0h (Fgf2-/-)*** | ***4h versus 0h***  ***(Fgf2+/+)*** | ***Fgf2-/- vs Fgf2+/+*** | ***P value*** | ***6h DMM versus naïve (Fgf2-/-)*** | ***6h DMM versus naïve (Fgf2+/+)*** | ***Fgf2-/- vs Fgf2+/+*** | ***P value*** |
| ***Acan*** | 1.4±0.4 | 1.2±0.4 (n.s) | 1.2 | ns | 0.73±0.14 | 0.9±0.2 (n.s) | 0.81 | n.s |
| ***Adam8*** | 1.6±0.8 | 1.8±0.6 (n.s) | 0.9 | ns | 5.8±1.4 | 3.6±0.1 (≤0.05) | 1.6 | ≤0.01 |
| ***Adam9*** | 0.3±0.4 | 1.5±0.3 (≤0.05) | 0.2 | ns | 2.8±0.7 | 3.1±0.1 (≤0.05) | 0.9 | n.s |
| ***Adamts1*** | 3.0±1.0 | 2.5±1.0 (≤0.05) | 1.2 | ns | 2.4±0.2 | 6.7±4.9 (≤0.05) | 0.4 | n.s |
| ***Adamts15*** | 0.2±0.2 | 0.2±0.1 (≤0.05) | 1.4 | ns | nd | nd | - | - |
| ***Adamts4*** | 1.4±0.4 | 1.3±0.5 (n.s) | 1.1 | ns | 4.9±0.5 | 4.5±0.5 (≤0.05) | 0.9 | n.s |
| ***Adamts5*** | 1.8±0.4 | 2.0±0.4 (≤0.05) | 0.9 | ns | 2.0±0.5 | 1.8±0.2 (≤0.05) | 1.1 | n.s |
| ***Ar*** | 0.8±0.2 | 0.5±0.2 (≤0.05) | 1.6 | ns | 7.6±4.2 | 2.9±1.5 (≤0.05) | 2.6 | ≤0.05 |
| ***Arg1*** | 7.6±6.0 | 113±93 (≤0.05) | 0.1 | ≤0.01 | 5.8±4.9 | 98.7±23.9 (≤0.05) | 0.05 | ≤0.001 |
| ***Arg2*** | 5.2±3.3 | 2.6±1.3 (≤0.05) | 2.0 | ns | 5.2±0.8 | 2.1±1.2 (≤0.05) | 2.5 | ≤0.001 |
| ***Ccl2*** | 17±15 | 12±7.9 (≤0.05) | 1.4 | ns | 12.0±3.0 | 153.3±42.3 (≤0.05) | 0.08 | ≤.001 |
| ***Ccl5*** | 1.6±0.9 | 1.8±1.1 (n.s) | 0.9 | ns | 1.5±0.1 | 1.6±0.3 (≤0.05) | 0.9 | n.s |
| ***Ccl7*** | 31±31 | 7.0±4.0 (≤0.05) | 4.4 | ≤0.01 | 22.6±6.8 | 29.8±1.1 (≤0.05) | 0.8 | ≤0.05 |
| ***Ccr2*** | 0.3±0.2 | 0.2±0.1 (≤0.05) | 1.3 | ns | 1.9±0.2 | 2.3±0.5 (≤0.05) | 0.8 | n.s |
| ***Ccr5*** | 1.3±0.7 | 1.4±0.7 (n.s) | 0.9 | ns | 2.0±1.1 | 3.2±0.4 (≤0.05) | 0.6 | ≤0.05 |
| ***Cd14*** | 6.8±0.6 | 16±6.7 (≤0.05) | 0.4 | ≤0.001 | 3.9±1.7 | 6.8±0.4 (≤0.05) | 0.6 | ≤0.01 |
| ***Cd68*** | 0.2±0.1 | 0.2±0.1 (≤0.05) | 1.2 | ns | 5.4±0.6 | 2.4±0.2 (≤0.05) | 2.25 | ≤0.001 |
| ***Col2a1*** | 1.1±0.3 | 1.2±0.6 (n.s) | 0.9 | ns | 0.5±0.2 | 0.5±0.2 (n.s) | 1.0 | n.s |
| ***Ctgf*** | 2.9±0.9 | 3.2±1.2 (≤0.05) | 0.9 | ns | 1.3±0.2 | 0.7±0.1 (n.s) | 1.8 | n.s |
| ***Esr1*** | 0.8 ±0.2 | 0.5±0.1 (≤0.05) | 1.5 | ns | 21.7±4.3 | 5.4±3.1 (n.s) | 4.0 | ≤0.001 |
| ***F3*** | 12±4.8 | 16±7.8 (≤0.05) | 0.8 | ns | 14.1±2.1 | 1.8±0.9 (≤0.05) | 7.8 | ≤0.001 |
| ***Has1*** | 6.9±3.4 | 17±9.9 (≤0.05) | 0.4 | ≤0.01 | 3.1±0.6 | 2.6±0.2 (≤0.05) | 1.2 | n.s |
| ***Has2*** | 2.1±0.4 | 2.7±1.5 (≤0.05) | 0.9 | ns | 0.9±0.02 | 2.0±0.8 (≤0.05) | 0.45 | ≤0.05 |
| ***Il1a*** | 6.2±5.8 | 3.2±1.7 (n.s) | 1.9 | ns | 2.1±0.1 | 1.3±0.2 (n.s) | 1.6 | ≤0.001 |
| ***Il1b*** | 12±11 | 4.1±1.0 (≤0.05) | 2.9 | ns | 5.4±1.0 | 5.6±0.4 (≤0.05) | 1.0 | n.s |
| ***Il1r1*** | 2.9±0.7 | 3.5±0.9 (≤0.05) | 0.8 | ns | 6.3±0.9 | 3.4±1.1 (≤0.05) | 1.9 | ≤0.001 |
| ***Il1rl1*** | 39±34 | 48±27 (≤0.05) | 0.7 | ns | 1.9±0.7 | 1.8±0.4 (≤0.05) | 1.1 | n.s |
| ***Il33*** | 1.2±0.6 | 2.2±1.4 (≤0.05) | 0.5 | ns | 3.5±1.1 | 2.6±0.6 (≤0.05) | 1.4 | n.s |
| ***Il6*** | 75±34 | 48±31 (≤0.05) | 1.6 | ns | 2.1±0.0 | 21.0±0.0 (≤0.05) | 0.1 | ≤0.001 |
| ***Inhba*** | 34±7.7 | 99±57 (≤0.05) | 0.4 | ≤0.01 | 0.5±0.1 | 2.6±0.4 (≤0.05) | 0.2 | ≤0.001 |
| ***Mmp13*** | 0.7±0.1 | 0.7±0.2 (n.s) | 1.0 | ns | 0.3±0.1 | 0.6±0.1 (n.s) | 0.5 | ≤0.05 |
| ***Mmp19*** | 0.9±0.3 | 3.4±1.8 (≤0.05) | 0.3 | ≤0.001 | 2.7±0.5 | 3.1±0.1 (≤0.05) | 0.9 | n.s |
| ***Mmp3*** | 2.6±1.2 | 3.3±1.0 (≤0.05) | 0.8 | ns | 2.9±0.7 | 6.0±0.3 (≤0.05) | 0.5 | ≤0.001 |
| ***Mmp8*** | 0.6±0.7 | 0.3±0.3 (≤0.05) | 2.1 | ns | 1.5±0.3 | 2.1±0.1 (≤0.05) | 0.7 | ≤0.01 |
| ***Nos2*** | 28±21 | 2.9±2.0 (≤0.05) | 9.5 | ≤0.001 | nd | nd | - | - |
| ***Pdpn*** | 3.0±1.0 | 5.9±1.3 (≤0.05) | 0.5 | ≤0.001 | 3.1±0.9 | 6.9±0.2 (≤0.05) | 0.5 | ≤0.001 |
| ***Ptges*** | 1.9±1.1 | 1.8±0.7 (≤0.05) | 1.1 | ns | 2.1±0.1 | 1.6±0.1 (≤0.05) | 1.3 | n.s |
| ***Ptgs2*** | 16±5.7 | 15.7±8.5 (≤0.05) | 1.0 | ns | 19.6±2.3 | 15.6±1.2 (≤0.05) | 1.3 | ≤0.01 |
| ***Serpina1a*** | 1.5±0.7 | 1.1±0.4 (n.s) | 1.4 | ns | 2.5±0.2 | 33.3±5.0 (≤0.05) | 0.1 | ≤0.001 |
| ***Sfrp2*** | 0.04±0.1 | 0.1±0.2 (≤0.05) | 0.3 | ns | 1.0±0.0 | 1.0±0.0 (n.s) | 1.0 | n.s |
| ***Timp1*** | 3.1±0.5 | 4.6±1.0 (≤0.05) | 0.7 | ns | 2.5±0.6 | 5.1±0.12 (≤0.05) | 0.5 | ≤0.001 |
| ***Tnfaip6*** | 13 ±2.5 | 40±22 (≤0.05) | 0.3 | ≤0.001 | 2.2±0.5 | 33.8±1.6 (≤0.05) | 0.1 | ≤0.001 |
| ***Tnfrsf12a*** | 7.6±3.9 | 12±6.7 (≤0.05) | 0.7 | ≤0.05 | 1.1±0.1 | 5.8±1.1 (≤0.05) | 0.2 | ≤0.001 |
| ***Wisp2*** | 0.5±0.14 | 0.6±0.2 (≤0.05) | 1.0 | ns | 3.4±0.2 | 7.0±0.1 (≤0.05) | 0.5 | ≤0.001 |
| ***Wnt16*** | 0.9±0.4 | 0.9±0.9 (n.s) | 1.0 | ns | 2.6±1.9 | 0.7±0.2 (n.s) | 3.7 | n.s |

Gene expression in wild type (fgf2+/+) and knockout (Fgf2-/-) tissue following in vitro and in vivo injury. For in vitro injury, hip explants were avulsed from 5 week old Fgf2+/+ or Fgf2-/- animals and cultured for 0 or 4h prior to RNA extraction. For in vivo experiments 10 week old male mice either had DMM surgery or were left unoperated (naïve) (data taken from [19]). 6h following surgery animals were culled and RNA extracted from whole joints (after skin and muscle had been removed). Genes were analysed using Taqman low density array microfluidic cards. Genes of interest were normalised to *18s* and expressed relative to the 0h or naïve samples. n=6 for all groups. Statistical analysis was by two-way ANOVA with Bonferroni post hoc testing, n.s = non-significant. n.d = not done. P values demonstrate level of significance. Acan – aggrecan; Ar – androgen receptor; Arg2 – arginase 2; Ccr – chemokine receptor; ctgf – connective tissue growth factor; Esr1 – estrogen receptor 1; F3 – tissue factor; Has – hyaluronan synthase; IL1rl – IL1 receptor like; Nos – nitric oxide synthase; Pges – prostaglandin synthase; Sfrp – secreted frizzled related protein; Tnfrsf12a – also known as TWEAK; Wisp – wnt inducible secreted protein.

Supplemental Table 2. Gene expression following stimulation of rested explants with FGF2 or FGF18.

| ***GENE*** | **FGF2 regulated? (from Table 2 except where indicated)** | **P value** | **Fold Change FGF18** | **P value** |
| --- | --- | --- | --- | --- |
| ***Adamts1*** | no |  | 1.7±0.2 | n.s |
| ***Adamts4*** | no |  | 1.2±0.24 | n.s |
| ***Adamts5*** | no |  | 1.6±0.18 | n.s |
| ***Arg1*** | no |  | 0.7±0.13 | n.s |
| ***Ccl2*** | no |  | 1.2±0.15 | n.s |
| ***Fgf18*** | 2.6±0.31 | ≤0.05 | 3.2±0.7 | ≤0.01 |
| ***Fgf2*** | 1.6±0.13 | n.s | 2.3±0.23 | n.s |
| ***Fgfr1*** | 1.0±0.12 | n.s | 1.5±0.22 | n.s |
| ***Fgfr2*** | 0.6±0.05 | n.s | 0.7±0.09 | n.s |
| ***Fgfr3*** | 0.4±0.03 | ≤0.001 | 0.4±0.05 | ≤0.001 |
| ***Il1b*** | no |  | 1.0±0.05 | n.s |
| ***Il6*** | no |  | 1.8±0.67 | n.s |
| ***Inhba*** | yes |  | 3.1±0.46 | ≤0.05 |
| ***Mmp13*** | no |  | 1.7±0.65 | n.s |
| ***Mmp3*** | no |  | 1.9±0.35 | n.s |
| ***Ptgs2*** | yes |  | 4.0±0.82 | ≤0.001 |
| ***Timp1*** | yes |  | 2.9±0.64* | n.s |
| ***Tnfaip6*** | yes |  | 3.1±0.37 | ≤0.05 |
| ***Tnfrsf12a*** | yes |  | 3.1±0.21 | ≤0.05 |

Hips were avulsed from 4-5 week old mice and rested in serum free medium for 48h. 6 hips were pooled and stimulated with FGF2 (20ng/ml), FGF18 (100ng/ml) or with fresh serum-free medium. After 4h mRNA was extracted and RT-PCR performed using Taqman low density array microfluidic cards. Genes of interest (as before as well as new FGF ligands and receptors) were normalised to *18s* and expressed relative to control samples (4h serum-free medium). n=3. Statistical analysis was by two-way ANOVA with Bonferroni post hoc testing, n.s = non-significant. P values demonstrate level of significance. Gene codes as above. *Significant regulation was seen on repeat using single primer RT-PCR.
